# A multilevel non-hierarchical study of birth weight and socioeconomic status

**DOI:** 10.1186/1476-072X-9-36

**Published:** 2010-07-09

**Authors:** Robin L Young, Janice Weinberg, Verónica Vieira, Ann Aschengrau, Thomas F Webster

**Affiliations:** 1 Department of Biostatistics, Boston University School of Public Health, Boston, MA, USA; 2 Department of Environmental Health, Boston University School of Public Health, Boston, MA, USA; 3 Department of Epidemiology, Boston University School of Public Health, Boston, MA, USA

## Abstract

**Background:**

It is unclear whether the socioeconomic status (SES) of the community of residence has a substantial association with infant birth weight. We used multilevel models to examine associations of birth weight with family- and community-level SES in the Cape Cod Family Health Study. Data were collected retrospectively on births to women between 1969 and 1983 living on Cape Cod, Massachusetts. The sample included siblings born in different residences with differing community-level SES.

**Methods:**

We used cross-classified models to account for multiple levels of correlation in a non-hierarchical data structure. We accounted for clustering at family- and community-levels. Models included extensive individual- and family-level covariates. SES variables of interest were maternal education; paternal occupation; percent adults living in poverty; percent adults with a four year college degree; community mean family income; and percent adult unemployment.

**Results:**

Residual correlation was detected at the family- but not the community-level. Substantial effects sizes were observed for family-level SES while smaller magnitudes were observed for community-level SES. Overall, higher SES corresponded to increased birth weight though neither family- nor community-level variables had significant associations with the outcome. In a model applied to a reduced sample that included a single child per family, enforcing a hierarchical data structure, paternal occupation was found to have a significant association with birth weight (p = 0.033). Larger effect sizes for community SES appeared in models applied to the full sample that contained limited covariates, such as those typically found on birth certificates.

**Conclusions:**

Cross-classified models allowed us to include more than one child per family even when families moved between births. There was evidence of mild associations between family SES and birth weight. Stronger associations between paternal occupation and birth weight were observed in models applied to reduced samples with hierarchical data structures, illustrating consequences of excluding observations from the cross-classified analysis. Models with limited covariates showed associations of birth weight with community SES. In models adjusting for a complete set of individual- and family-level covariates, community SES was not as important.

## Background

Birth weight is a measure of overall infant health and a predictor of death and long-term disability [1]. Previous research has shown that family socioeconomic status (SES) is positively associated with birth weight; [2,3] however after adjusting for this and other known predictors, substantial variability in birth weight remained. Increased rates of low birth weight (< 2500 g) have been observed in impoverished areas [4-6] leading to the hypothesis that community-level socioeconomic factors also influence birth weight [7,8]. Contextual effects on health by community of residence may be mediated by health service availability, shared attitudes towards health care, and sources of chronic stressors and social supports [6]. To understand these complex relationships, previous studies examined associations of birth weight with community-level SES measures such as unemployment rate, per capita or family income, percent adults with lower levels of education, and percent adults living in poverty [3,4,8].

Researchers turned to multilevel models to examine the impact of family- and community-level SES on birth weight while accounting for residual community level correlation between subjects [3,4,9,10]. In these studies families were "nested" within neighborhoods and samples included one child per family. Two levels were examined: the child-/family-level, including child and family characteristics, and the community-level, including community SES. Previous literature measured varied magnitudes of association, and variability in child- and family-level confounders in multivariate analyses led to inconsistent conclusions. Studies finding effects of community SES tended to adjust for limited covariates available from birth certificate or registry reviews [3,4,8]. Noticeably absent from these analyses were adjustments for gestational duration and maternal smoking status during pregnancy. Morenoff et al. found that the effects of community variables observed in crude analyses were reduced when adjusting for an extensive list of child and family characteristics, [9] suggesting that observed community-level effects may be a consequence of unmeasured confounding.

Traditional multilevel models were limited by the requirement of a single child per household. If siblings born in the same community were included in the sample a third level would be introduced to the multilevel analysis: the child-level, family-level, and community-level. The model would assume children were nested within families and families were nested within communities [11]. Models of this structure are straightforward to consider and can be applied in a number of software programs.

However, it is not reasonable to assume that the family lived in the same community for all births. Relocation is relatively common as 47% of United States residents reported relocation between 1965 and 1970 [12] and, in 1988, 74% of a nationally representative sample of children in the United States had moved residential location at least once in their lifetime [13]. Different family residences may lead to sibling differences in community SES and other characteristics at birth. Nested hierarchical models cannot adequately represent the study population. While they are uncommon in current birth weight research, cross-classified models are useful in analyzing data with non-hierarchical structures [14]. Cross-classified multilevel statistical models should be used to consider families that may have moved from one community to another between child births. These models allow for correlation between siblings as well as between children born in the same community. Cross-classified models can be expressed similarly to hierarchical models in mathematical notation [14] and can be analyzed through available statistical software such as SAS [15].

The purpose of this paper is three-fold. The first objective is to evaluate the importance of family- and community-level measures of SES in predicting birth weight in the Cape Cod Family Health Study, a retrospective cohort study conducted on Cape Cod, Massachusetts. This study provides an example of a non-hierarchical data structure with siblings born in different communities. To the best of our knowledge, no previous research has examined the multilevel association between family- and community-level SES and birth weight allowing for a non-nested data structure. The second objective is instructive as we illustrate how such non-hierarchical data can be appropriately analyzed and give recommendations for the analysis of multilevel data in general. As we collected extensive individual- and family-level information about our study participants, our third objective is to compare the results of multilevel models using full confounder information with those of models including limited data typically found in birth records. From these analyses we are able to assess confounding of birth weight associations with SES in our study population.

## Methods

### Study Population

The Cape Cod Family Health Study is a retrospective cohort study designed to investigate the relationship between environmental PCE (perchloroethylene, tetrachloroethylene) exposure from contaminated drinking water and reproductive health outcomes on Cape Cod, Massachusetts. Children were eligible for the study if they were born between 1969 and 1983 to a mother living in one of eight Cape Cod towns [16]. We restricted the present analyses to full term infants (gestation ≥ 37 weeks) to eliminate the impact of prematurity on birth weight. The Institutional Review Boards at Boston University Medical Center and the Massachusetts Department of Public Health approved this research.

These data provided an opportunity to examine predictors of birth outcomes at child-, family-, and community-levels. Current analyses focused on birth weight, obtained from birth certificates. We selected continuous birth weight as it provides an opportunity to apply cross-classified models to a continuous outcome. Child- and family-level data were ascertained through birth certificate review and self-administered questionnaires sent to the mothers in 2002-2003. For community-level variables, study mothers' residential addresses at time of birth were linked to census data from 1980. These data were closest to the birth years of children in the study: 89% were born between 1974 and 1983. Community-level data was purchased from GeoLytics (East Brunswick, NJ) at the enumeration district level. Enumeration districts are roughly the same size as census tracts and were used to designate communities as Cape Cod was not divided into census tracts until after 1980 [17].

Predictors of interest included family- and community-level SES variables. At the family-level, SES was measured by maternal education (less than high school, high school graduate, or some college) and paternal occupation (white collar, blue collar, or other) at time of birth. Community-level SES measures included percent adults living in poverty, mean family income, percent adults graduated from a four-year college, and percent unemployment in the enumeration district of residence. Mean family income and percent adults graduated from a four-year college were parameterized by quartiles while percent adults living in poverty and percent unemployment were dichotomized at 0 and 10%, respectively, based on evidence of a clear cut point from an unadjusted locally-weighted running-line smoothing (LOESS) plot [18].

Other confounding variables were selected based on known relevance to birth weight. From birth certificate review we included child gender, gestational duration (> = 37 weeks), birth order, year of birth, maternal age, and adequacy of prenatal care. From survey questionnaires we obtained information on the adequacy of maternal weight gain, maternal history of cervical incompetence, and smoking and alcohol consumption during pregnancy. Family-level covariates included maternal race from birth certificate review, maternal history of diabetes or hypertension before or during pregnancy and history of prior low birth weight (birth weight < 2500 g) or premature (gestation < 37 weeks) infants from the questionnaire.

Differences between family-level SES measures could occur between siblings due to changes in paternal occupation, maternal education, or paternity. Differences between community-level SES measures could occur due to family relocation between child births.

### Statistical Analysis

Smooth LOESS plots [18] were used to examine unadjusted relationships between continuous group-level SES measures and birth weight. Cut points identified in these plots were used to create categories for analyses.

We expected residual correlation within families and enumeration districts as siblings likely have correlated birth weights after adjusting for other predictors due to genetic similarities. Children within enumeration districts may also have comparable birth weights due to similar parental behaviors or environmental exposures. We used a mixed model with random intercepts for family and enumeration district to account for these correlations.

The mixed model was non-hierarchical due to cross-classification of sibling births between enumeration districts. The applied model was a mixed linear regression model as follows:

*Y*_*ijk *_represented the birth weight of the *k*^*th *^child from the *j*^*th *^family living in the *i*^*th *^enumeration district. The term *α *represented the intercept, *X*_*k *_the child- and family-specific demographic characteristics, and *W*_*i *_and *V*_*j *_represented the community- and family-level SES values, respectively. The corresponding regression coefficients were *β*, *λ*, and *γ*. *d*_*i *_and *f*_*j *_were random intercepts for enumeration district and family [14]. Formally, this allowed correlation within families and enumeration districts, resulting in variation in mean birth weight at two levels. For simplicity, the model ignored any possible spatial correlation due to proximity of enumeration districts.

Three mixed linear regression models were compared to evaluate the necessity of random intercepts. Model I included random intercepts for enumeration district only, Model II had random intercepts for family only and Model III allowed for variation between both family and enumeration district means. It was assumed that family and enumeration district effects were distinct and the variances of each level could be identified. (Table 1) Hypothesis tests were performed for each of the models to evaluate whether the variance of the random intercepts were non-zero. Akaike's Information Criteria (AIC), a measure of model fit, was compared [19].

**Table 1:** Model Descriptions

Model	Model Type	Random Effects	Variables Included/Excluded	Sample Restrictions
Model I	Mixed Model	Enumeration district	Full model	Full sample

Model II	Mixed Model	Family	Full model	Full sample

Model III	Cross-classified Model	Enumeration district, Family	Full model	Full sample

Model IIIa	Cross-classified Model	Enumeration district, Family	Excluding family-level SES measures	Full sample

Model IIIb	Cross-classified Model	Enumeration district, Family	Excluding community-level SES measures	Full sample

Model IV	Mixed Model	Enumeration district	Full model	Restricted to eldest child in family

Model V	Hierarchical Model	Enumeration district, Family	Full model	Restricted to later-born children born in same enumeration district as eldest child in family (nested data hierarchy)

Model VI	Cross-classified Model	Enumeration district, Family	Including covariates available on birth certificate only	Full sample

Model VIa	Cross-classified Model	Enumeration district, Family	Including covariates available on birth certificate and gestational duration	Full sample

Model VIb	Cross-classified Model	Enumeration district, Family	Including covariates available on birth certificate and maternal smoking status during pregnancy	Full sample

We applied two additional cross-classified models to evaluate possible confounding of family- and community-level SES measures. Model IIIa excluded family-level SES variables while Model IIIb excluded community-level SES variables. (Table 1)

Two models were performed using restricted samples to impose a hierarchical data structure and avoid the need for cross-classified models. For Model IV, the sample was restricted to the eldest child per family. Random intercepts for enumeration district were included. Model V allowed multiple children per family but required that all children be born in the same enumeration district creating a nested hierarchy. In this sub-sample, later-born children were considered eligible if they were born in the same enumeration district as the eldest child. Random intercepts were included for both family and enumeration district.

Predictors of primary interest were family- and community-level SES measures. All analyses were adjusted for relevant covariates. We tested for confounding by PCE exposure but found no association with the outcome. As a result, the models presented do not control for PCE exposure.

We analyzed a birth certificate covariates only model (Model VI) to test for association between community-level SES and infant birth weight adjusting only for variables selected from birth certificate review. Variables reflected those used in the previous research that found significant associations between community SES and birth outcomes: child birth order, gender, birth year, adequate prenatal care, maternal age, maternal race, and maternal education [3,4,8]. To evaluate confounding, Model VI was compared to two models including either gestational duration (Model VIa) or maternal smoking status during pregnancy (Model VIb) in addition to the variables listed above. We chose these comparisons as they are known important risk factors for low birth weight and gestation was often excluded from prior studies [3,4,8] and, at the time these data were collected, maternal smoking status during pregnancy was not available from Massachusetts birth certificates [20].

All analyses were performed using SAS Version 9.1 [15]. (Example SAS code to apply cross-classified models is available in Additional file 1.) Beta coefficients were used to assess the magnitude of the effect of SES on birth weight and p-values were used to assess the precision of the coefficients. All hypothesis tests were performed with a significance level of 0.05.

## Results

The Cape Cod Family Health Study consisted of 2,144 subjects. Subjects included in this analysis were restricted to full term singletons born after 1975 without congenital anomalies whose mothers responded to the self-administered questionnaire. (n = 1,689) Subjects with at least one missing covariate were excluded from analyses. (n = 240) No single covariate had more than 5% missing values. The final sample included 1,449 children from 1,252 families living in one of 170 enumeration districts. Of those families, 175 had between two and four children in the study and 34 families moved between enumeration districts at least once between births. On average, enumeration districts included 9 children, with a range of 1 to 79. There was evidence of variation in birth weight: the sample mean was 3524.0 g and the standard deviation was 466.4 g. The standard deviation of family mean birth weight was 460.0 g while the standard deviation of mean birth weight in enumeration districts was smaller (247.8 g). This is a crude illustration that enumeration districts accounted for less variation in birth weight than families.

Infants were nearly 50% female with an average gestational duration of 40.2 weeks. Fewer than 5% of mothers had a history of giving birth to a low birth weight infant, 8% had a history of hypertension, and over 95% of mothers were white. Over one-fourth of mothers smoked during pregnancy and nearly 40% consumed alcoholic beverages, both behaviors have been shown to decrease birth weight [21,22] (Table 2).

**Table 2:** Sample Demographic Characteristics

Characteristic	N	%
Child Birth Weight (Mean ± SD)	3524.0 ± 466.4	
Infant Gender		
Male	1081	(50.4)
Female	1063	(49.6)
Gestational Duration (Mean ± SD)	40.2 ± 2.1	
< 37 weeks	97	(4.5)
37-41 weeks	1633	(76.2)
> 41 weeks	395	(18.4)
Missing	19	(0.9)
Birth Order		
1	827	(38.6)
2	766	(35.7)
3+	520	(24.3)
Missing	31	(1.4)
Year of Birth		
1969-1973	248	(11.6)
1974-1978	695	(32.4)
1979-1983	1201	(56.0)
Adequate Prenatal Care		
Yes	1546	(72.1)
No	212	(9.9)
Missing	386	(18.0)
Inadequate Weight Gain		
Yes	179	(8.3)
No	1858	(86.7)
Missing	107	(5.0)
Maternal Age (Mean ± SD)	27.5 ± 4.5	
Maternal Race		
White	2062	(96.2)
Non-White	82	(3.8)
History of Diabetes		
Yes	66	(3.1)
No	2027	(94.5)
Missing	51	(2.4)
History of Hypertension		
Yes	159	(7.4)
No	1930	(90.0)
Missing	55	(2.6)
Cervical Incompetence		
Yes	42	(2.0)
No	2073	(96.6)
Missing	29	(1.4)
Prior Low Birth Weight Infant		
Yes	94	(4.4)
No	2008	(93.7)
Missing	42	(2.0)
Prior Preterm Infant		
Yes	128	(6.0)
No	1963	(97.5)
Missing	53	(2.5)
Mother Smoked During Pregnancy		
None	1526	(71.2)
Average 1-10 cigarettes per day	289	(13.5)
Average 11-20 cigarettes per day	206	(9.6)
Average 20 + cigarettes per day	83	(3.9)
Missing	40	(1.9)
Mother Consumed Alcohol During Pregnancy		
None	1256	(58.6)
During 1-2 trimesters	209	(9.8)
During 3 trimesters	615	(28.7)
Missing	64	(3.0)
Maternal Education		
< HS Education	78	(3.6)
HS Graduate	763	(35.6)
Some College	1302	(60.7)
Missing	1	(0.0)
Paternal Occupation		
White collar	1054	(49.2)
Blue collar	694	(32.4)
Service occupations	359	(16.7)
Missing	37	(1.7)

Over 60% of mothers had at least a high school degree and half of fathers had white collar occupations. (Table 2) 14% of enumeration districts had some adults living in poverty. The mean enumeration district family income was over $19,500 with a standard deviation of over $6,000. An enumeration district-level mean of 14% of adults had a four-year college degree with an average of 8% unemployed. (Table 3)

**Table 3:** Enumeration District Characteristics

Characteristic	N	%
% Adults living in Poverty (Mean ± SD)	14.0 ± 14.0	
0%	62	(34.4)
> 0%	115	(63.9)
Missing	3	(1.7)
Mean Family Income (Mean ± SD)	19542.6 ± 6202.3	
Quartile 1	57	(31.7)
Quartile 2	43	(23.9)
Quartile 3	22	(12.2)
Quartile 4	55	(30.6)
Missing	3	(1.7)
% Adults w/4 Year College Degree (Mean ± SD)	14.0 ± 8.9	
Quartile 1	41	(22.8)
Quartile 2	50	(27.8)
Quartile 3	27	(15.0)
Quartile 4	59	(32.8)
Missing	3	(1.7)
% Adults Unemployed (Mean ± SD)	8.1 ± 7.1	
< 10%	134	(74.4)
≥ 10%	43	(23.9)
Missing	3	(1.7)

**Table 4:** Partitioning of Variance by Model Level for Cross-Classified Models

	Model I	Model II	Model III
	Variance (P-Value)	Variance (P-Value)	Variance (P-Value)
Enumeration District	19.78(0.4950)		129.21(0.4659)
Family		99522.21(< 0.001)	99447.2(< 0.001)

Residual	179433.79(< 0.001)	81259.62(< 0.001)	81225.56(< 0.001)

Of families moving from one enumeration district to another, 7% of mothers increased their education between births and 30% of fathers changed occupation categories. For community-level SES, 13% moved to areas with some adults living in poverty and 23% moved from these communities; 76% changed mean family income quartile, and 80% changed percent of adults with a college degree. 17% of families moved into enumeration districts with more than 10% adult unemployment and 13% moved away from these areas.

### Random Effects

In Model III, the residual correlation was 0.5507 for siblings born in the same enumeration district, 0.5504 for siblings born in different enumeration districts, and 0.0016 for unrelated subjects born in the same enumeration district. Unrelated subjects born in different enumeration districts were assumed to be independent.

Based on tests of non-zero variance for random intercepts, the best fit to the data was observed in Model I, a conclusion supported by comparison of AIC statistics. In circumstances where a random effect is not statistically significant, researchers typically select the most parsimonious model for further analyses. Here there was no difference in interpretation as residual correlation within enumeration districts was near zero. For instructive purposes we interpreted Model III, the cross-classified model including random intercepts for family and enumeration district.

### Family- and Community-Level SES

In Model III, mothers with some college education gave birth to infants weighing 128 g more than mothers with less than a high school diploma while smaller effect sizes were observed for other SES variables. (Table 5)

**Table 5:** Fixed Effects for Cross-Classified Models

		Model III	Model IIIa	Model IIIb
		β (grams) (SE;P-Value)	β (grams) (SE;P-Value)	β (grams) (SE;P-Value)
	**AIC**	**21201.52**	**21243.31**	**21279.45**

Gender	Male v Female	158.30(21.62; < 0.001)	159.07(21.57; < 0.001)	160.25(21.60; < 0.001)
Gestational duration	Weeks	52.94(6.49; < 0.001)	51.25(6.46; < 0.001)	54.34(6.47; < 0.001)
Birth order	-	< 0.001	< 0.001	< 0.001
	1v3+	-229.17(34.44; < 0.001)	-221.10(33.44; < 0.001)	-223.98(34.35; < 0.001)
	2v3+	-145.33(31.21; < 0.001)	-141.77(30.69; < 0.001)	-139.72(31.13; < 0.001)
Year of birth		0.094	0.093	0.150
	(1975 v 1983)	-63.72(53.91;0.239)	-63.95(53.91;0.238)	-69.04(53.75;0.201)
	(1976 v 1983)	61.63(54.52;0.260)	62.94(54.34;0.249)	46.96(54.30;0.389)
	(1977 v 1983)	-61.82(47.24;0.193)	-63.26(47.24;0.183)	-60.90(47.10;0.198)
	(1978 v 1983)	-40.11(44.6;0.370)	-40.20(44.55;0.368)	-45.58(44.56;0.308)
	(1979 v 1983)	-63.04(41.19;0.128)	-67.56(41.16;0.103)	-64.65(41.14;0.118)
	(1980 v 1983)	37.05(39.51;0.350)	31.98(39.41;0.418)	29.39(39.39;0.457)
	(1981 v 1983)	-1.26(41.97;0.976)	-4.23(41.93;0.920)	-3.79(41.88;0.928)
	(1982 v 1983)	-4.98(41.07;0.904)	-9.10(41.04;0.825)	-9.10(41.01;0.825)
Adequate prenatal care	Yes v No	73.98(35.73;0.038)	82.16(35.58;0.021)	71.07(35.66;0.046)
Inadequate weight gain	Yes v No	-184.84(44.11; < 0.001)	-190.94(44.02; < 0.001)	-181.72(44.06; < 0.001)
Maternal age	Years	-3.08(3.28;0.348)	-1.91(3.07;0.534)	-2.12(3.26;0.516)
Maternal race	White v NonWhite	188.19(65.56;0.004)	199.20(65.35;0.002)	189.53(65.20;0.004)
Any diabetes	Yes v No	246.00(70.70;0.001)	235.57(70.66;0.001)	242.26(70.33;0.001)
Any hypertension	Yes v No	-17.51(45.94;0.703)	-15.25(45.89;0.740)	-23.49(45.72;0.607)
Cervical incompetence	Yes v No	-151.88(89.70;0.090)	-153.72(89.71;0.087)	-149.53(89.48;0.095)
Prior low birth weight infant	Yes v No	-245.43(64.48; < 0.001)	-237.67(64.55; < 0.001)	-248.83(64.17; < 0.001)
Prior preterm infant	Yes v No	-14.96(56.14;0.790)	-19.73(56.23;0.726)	-13.08(55.89;0.815)
Smoking category		< 0.001	< 0.001	< 0.001
	Avg < 10 cig v None	-140.06(34.18; < 0.001)	-148.57(33.99; < 0.001)	-141.18(34.10; < 0.001)
	Avg 11-20 cig v None	-212.59(43.57; < 0.001)	-219.62(43.41; < 0.001)	-212.27(43.39; < 0.001)
	Avg 21+ cig v None	-200.75(69.52;0.005)	-221.78(69.02;0.002)	-209.73(69.21;0.003)
Alcohol consumption		0.945	0.954	0.933
	> = 1 Trimester v None	-9.76(37.68;0.796)	-9.53(37.53;0.800)	-11.34(37.57;0.763)
	3 Trimesters v None	-7.59(27.92;0.786)	-6.29(27.83;0.821)	-7.88(27.84;0.777)

Maternal education		0.242		0.251
	< HS v College	-127.67(79.49;0.111)		-129.77(79.30;0.104)
	HS Grad v College	3.31(27.04;0.903)		-2.28(26.84;0.932)
Paternal occupation		0.102		0.085
	Blue v White Collar	-46.64(27.35;0.09)		-48.82(27.16;0.075)
	Other v White Collar	22.53(35.25;0.524)		21.88(35.18;0.535)

% Adults living in poverty	> 0% v 0%	-51.41(28.13;0.068)	-50.34(28.01;0.072)	
Mean family income		0.831	0.851	
(quartiles)	Q1vQ4	-3.13(37.61;0.934)	-10.38(37.33;0.781)	
	Q2vQ4	-0.52(35.04;0.988)	0.33(34.87;0.992)	
	Q3vQ4	-29.79(37.22;0.425)	-28.11(37.03;0.449)	
% Adults graduated		0.138	0.124	
4 year college (quartiles)	Q1vQ4	-50.49(37.06;0.175)	-51.99(36.8;0.16)	
	Q2vQ4	-0.53(34.55;0.988)	-3.00(34.41;0.931)	
	Q3vQ4	41.22(37.69;0.276)	41.29(37.52;0.273)	
% Unemployment	≤ 10% v > 10%	19.5(32.54;0.549)	18.84(32.37;0.561)	

^‡ ^Type III p-value for overall variation between categories

To understand whether the family- and community-level SES measures were independent of one another, we excluded the family-level SES measures from the analysis (Model IIIa). The estimates for some community-level SES measures changed by more than 10% suggesting confounding of the community-level effects by the two family-level measures; however the magnitudes of the effects remained small. When the community-level SES variables were excluded from the model (Model IIIb) there were small change to the coefficients of the family-level SES measures indicating little confounding by the community-level measures. (Table 5)

### Comparison to Sub-Samples with Nested Hierarchies

Model IV was applied to the eldest eligible child per family, excluding 262 later-born children from the analysis. Model V was applied to eligible eldest children and later-born children born in the same enumeration district as the eldest child. (n = 1,399, 50 subjects excluded) Increased maternal education effect sizes were observed for Models IV and V when compared to Model III. Most notably, the effect size was increased considerably for the comparison of women without a high school diploma to those who have attended some college for the models applied to sub-samples. In Model IV, paternal occupation was significantly associated with birth weight. (p = 0.033) Fathers with blue collar occupations had children with significantly lower birth weights than white collar occupations (p = 0.024) while no difference was detected between white and "other" occupations. (p = 0.651) Paternal occupation in Model V had a p-value just over the 5% cut-off, (p = 0.057) indicating a possible trend in the same direction as Model IV. Similar effect sizes were observed for all community-level SES variables in Models III, IV, and V. (Table 6)

**Table 6:** Fixed Effects for Family- and Community-Level SES for Model III, Model IV, and Model V

		Model III*	Model IV*^†^	Model V*^‡^
		β (grams) (SE;P-Value)	β (grams) (SE;P-Value)	β (grams) (SE;P-Value)
Maternal education		0.242	0.115	0.224
	< HS v College	-127.67(79.49;0.111)	-173.26(87.42;0.040)	-131.78(82.56;0.110)
	HS Grad v College	3.31(27.04;0.903)	4.87(29.34;0.868)	7.8(27.42;0.776)
Paternal occupation		0.102	0.033	0.057
	Blue v White Collar	-46.64(27.35;0.090)	-68.05(30.16;0.024)	-53.36(27.99;0.059)
	Other v White Collar	22.53(35.25;0.524)	17.62(38.96;0.651)	26.45(36.44;0.469)

% Adults living in poverty	> 0% v 0%	-51.41(28.13;0.068)	-37.79(30.8;0.220)	-49.95(29.37;0.089)
Mean family income (quartiles)		0.831	0.800	0.826
	Q1vQ4	-3.13(37.61;0.934)	-16.66(41.56;0.689)	-15.72(39.22;0.689)
	Q2vQ4	-0.52(35.04;0.988)	3.69(38.49;0.924)	-4.38(36.41;0.904)
	Q3vQ4	-29.79(37.22;0.425)	-31.80(41.31;0.442)	-33.5(39.03;0.392)
% Adults graduated 4 year college (quartiles)		0.138	0.555	0.335
	Q1vQ4	-50.49(37.06;0.175)	-25.81(41.19;0.531)	-25.06(38.76;0.519)
	Q2vQ4	-0.53(34.55;0.988)	13.9(38.40;0.717)	20.37(36.24;0.575)
	Q3vQ4	41.22(37.69;0.276)	34.05(41.32;0.410)	46.16(39.14;0.24)
% Unemployment	≤ 10% v > 10%	19.5(32.54;0.549)	18.97(36.84;0.607)	10.77(34.25;0.753)

*Models adjusted for child gender, gestational duration, birth order, year of birth, maternal age and race, adequate prenatal care, inadequate maternal weight gain, and cervical incompetence during pregnancy, any history of maternal diabetes or hypertension, prior low birth weight or preterm infants, and maternal smoking during pregnancy.

^†^Sub-sample included eldest child per family only (n = 1,187).

^‡^Sub-sample included all siblings born in same enumeration district as eldest child (n = 1,399).

### Birth Certificate Covariate Only Models

In Model VI, containing child gender, birth order, year of birth, adequacy of prenatal care, maternal age, and maternal race in addition to family- and community-level SES measures, percent of adults with a four year college education and maternal educational level had the strongest associations with birth weight. (Table 7)

**Table 7:** Fixed Effects for Models with Reduced Covariates

	Level	Model IV	Model IVa	Model IVb
		β (grams) (P-Value)	β (grams) (P-Value)	β (grams) (P-Value)
	**AIC**	**21594.53**	**21519.07**	**21515.5**

Gender	Male v Female	142.89(22.36; < 0.001)	155.81(22.04; < 0.001)	147.05(22.10; < 0.001)
Gestational duration	Weeks		56.23(6.60; < 0.001)	
Birth order	-	< 0.001	< 0.001	< 0.001
	1v3+	-169.84(34.43; < 0.001)	-178.72(33.76; < 0.001)	-182.39(34.10; < 0.001)
	2v3+	-115.09(31.58; < 0.001)	-102.30(31.17;0.001)	-130.50(31.37; < 0.001)
Year of birth		0.021	0.018	0.025
	(1975 v 1983)	-80.33(55.82;0.152)	-83.91(54.71;0.127)	-85.40(55.12;0.123)
	(1976 v 1983)	50.70(56.64;0.372)	39.70(55.55;0.476)	60.70(55.88;0.279)
	(1977 v 1983)	-67.24(48.62;0.169)	-85.48(47.84;0.076)	-49.98(48.21;0.302)
	(1978 v 1983)	-43.56(46.13;0.347)	-65.23(45.43;0.153)	-29.49(45.63;0.519)
	(1979 v 1983)	-76.52(42.42;0.073)	-78.76(41.77;0.061)	-67.50(42.02;0.110)
	(1980 v 1983)	43.95(40.58;0.281)	35.45(40.13;0.378)	50.35(40.25;0.213)
	(1981 v 1983)	10.01(43.24;0.817)	-0.34(42.71;0.994)	17.47(42.83;0.684)
	(1982 v 1983)	21.80(42.41;0.608)	6.61(41.77;0.874)	24.56(41.94;0.559)
Adequate prenatal care	Yes v No	108.62(37.24;0.004)	99.59(36.52;0.006)	95.28(36.76;0.010)
Maternal age	Years	-4.10(3.44;0.233)	-2.86(3.34;0.391)	-4.95(3.37;0.142)
Maternal race	White v NonWhite	211.69(67.95;0.002)	197.4(65.89;0.003)	243.75(66.73; < 0.001)

Smoking category				< 0.001
	Avg < 10 cig v None			-150.15(34.85; < 0.001)
	Avg 11-20 cig v None			-248.18(45.08; < 0.001)
	Avg 21+ cig v None			-254.02(71.42;0.001)
Maternal education		0.229	0.092	0.624
	< HS v College	-137.77(82.55;0.097)	-176(80.60;0.031)	-61.88(82.02;0.452)
	HS Grad v College	0.64(28.28;0.982)	-10.35(27.55;0.708)	11.80(27.81;0.672)
Paternal occupation		0.116	0.033	0.243
	Blue v White Collar	-56.66(28.59;0.049)	-66.44(27.87;0.018)	-43.24(28.16;0.127)
	Other v White Collar	-2.81(36.96;0.939)	7.33(36.07;0.839)	5.01(36.39;0.891)

% Adults living in poverty	> 0% v 0%	-43.96(29.48;0.136)	-39.04(28.66;0.173)	-52.10(28.97;0.072)
Mean family income (quartiles)		0.945	0.914	0.957
	Q1vQ4	-11.86(39.32;0.763)	-20.49(38.25;0.593)	-7.88(38.64;0.839)
	Q2vQ4	0.59(36.71;0.987)	-5.86(35.66;0.870)	1.29(36.02;0.971)
	Q3vQ4	-19.08(39.04;0.626)	-22.55(37.96;0.553)	-17.43(38.35;0.65)
% Adults graduated		0.019	0.094	0.054
4 year college (quartiles)	Q1vQ4	-81.91(38.54;0.035)	-66.80(37.54;0.077)	-67.30(37.92;0.078)
	Q2vQ4	-11.96(36.26;0.742)	-15.48(35.28;0.661)	-1.58(35.64;0.965)
	Q3vQ4	44.29(39.57;0.265)	30.93(38.49;0.423)	41.58(38.85;0.286)
% Unemployment	≤ 10% v > 10%	26.84(34.02;0.430)	30.37(33.11;0.359)	23.33(33.43;0.485)

^‡^Type III p-value for overall variation between categories

Inclusion of gestational duration, a confounder even after restricting the sample to full term infants, produced large changes in the coefficients for group-level SES. Similar results were observed when we added maternal smoking status during pregnancy to the model. (Table 7) In none of these models was it important to account for residual correlation at the community-level.

## Discussion

A positive trend of increased child birth weight for maternal completion of higher education was observed, consistent with previous research associating maternal education with birth outcomes [3,4]. For models applied to the full sample, paternal occupation did not have a significant association with birth weight. A large variety of positions held at the military base on Cape Cod, full time students, and other occupations were classified as "other", complicating the interpretation of this variable. Community-level SES showed little association with birth weight in the full model. This suggests little contextual effect of SES in this study population.

Strengths of this study include the relatively large sample size and collection of birth weight data from birth certificates and confounding variables from birth records and parental report. Due to the retrospective study design, subjects may have made errors in recalling and reporting past behaviors. While this may have affected responses to risk factors such as smoking and alcohol consumption during pregnancy, community-SES measures were obtained from 1980 census information and both birth weight and family-SES measures were obtained from birth certificates. The effects of community- and family-SES measures on birth weight should be unaffected by recall errors.

Community SES measures were based on enumeration district of residence at approximate time of child birth. The boundaries of enumeration districts may not adequately reflect groupings of families with latent similarities. Smaller geographic resolution may produce groups of families that were more similar than those available from enumeration districts. Although we previously found contextual effects of SES on risk of breast cancer on Cape Cod, [17] the area may be too homogenous to show strong effects of community SES on birth weight. Further studies in more diverse geographical areas are needed. SES measures selected for this analysis may not adequately capture SES at the community-level; however there are no standard methods for community SES assessment.

A study examining birth outcomes in the Chicago area in the mid-1990s where communities were defined as one or more geographically contiguous census tracts also found minimal community-level SES effects on birth weight after adjustment for individual- and family-level characteristics, including maternal education, marital status, and smoking and alcohol consumption during pregnancy as well as biomedical characteristics such as anemia, diabetes, previous low birth weight infants, and previous pregnancy terminations. Community SES measures such as percent of families in poverty and residential stability (a composite measure of owner occupied housing and residents living in a home for extended periods of time) had coefficient magnitudes of only 3.15 and 3.29 g per one unit increase in the corresponding variables when predicting birth weight. In analyses adjusted for fewer covariates these variables had coefficient magnitudes greater than 20 and 10 g per one unit increase, respectively. Interestingly, community-level variables capturing stress in the neighborhood, violent crime rate and reciprocated mutual support were more important predictors than percent of families living in poverty or residential stability with coefficient magnitudes greater than 10 g per unit increase in a model with limited covariates and magnitudes greater than 6 g per unit increase in a full model [9]. When predicting very low birth weight (< 1500 g) among U.S. born non-Hispanic white and African American women who delivered in New York City, there were no meaningful effects of community poverty level but individual-level factors were influential. There was an important effect of community poverty when predicting moderately low birth weight (1500-2500 g) [10]. Luo et al. found that community-level SES measures had only moderate association with birth outcomes (preterm birth, small for gestational duration, stillbirth, neo- and post-natal deaths) while individual SES factors had far greater effects [4]. Other studies found independent effects of community SES and individual-factors on child birth weight [3,8]. When stratified by ethnicity, the directions and magnitudes of association varied considerably between groups [8].

When we compared the individual-level factors included in previous studies, we found that studies with moderate to large associations between community-level SES and birth outcomes adjusted for a small number of individual-level covariates. These papers focused on community variables and did not control for known risk factors and possible mediators such as gestational duration and smoking status during pregnancy [3,4,8]. We analyzed comparable models including birth certificate covariates only and found similar results. Prior to adjusting for gestational duration or maternal smoking status, community SES had a significant association with birth weight (p = 0.019). Once adjusted for either one of these additional confounders, the magnitudes of coefficients decreased for two of three categories and there was no longer evidence of an association. Morenoff et al. also found reduced effects of community SES measures predicting low birth weight when adjusting for more individual characteristics than are available in birth records [9]. Rauh et al. found no meaningful association between community SES and very low birth weight when controlling for maternal smoking status, substance use during pregnancy, marital status, maternal education, and child birth order though some associations were seen for moderately low birth weight [10]. Associations observed between birth weight and community SES may have resulted from residual confounding from individual-level characteristics.

No previous study allowed or accounted for multiple children per family. Statistical models assumed either nested data [3,8-10] or independent observations after determination of negligible correlation at the community-level [4]. Data in this study included multiple children per family and changes of residential address between births. When we restricted this sample to a single child per family we observed increased effect sizes for family-level SES variables. While inferences from Models III, IV, and V were otherwise very similar, it is noteworthy that conclusions based on family-level SES would have differed had we restricted our sample. The cross-classified model allowed us to include all observations, an advantage that may affect inferences, particularly for highly mobile populations. It may also improve representativeness of the sample when compared to the entire population. As was appropriate for a non-nested data structure, we adjusted for family- and community-level SES and accounted for multiple sources of correlation using a cross-classified statistical model using standard software.

When drawing samples from populations with a primary interest in community-level variables, subjects often fall into subgroups within communities. In the present study, subgroups were families. We found that sibling birth weights were highly correlated and between-family variation accounted for much of the overall birth weight variation. Incorrectly assuming that subjects are independent may lead to incorrect variability estimates and test statistics.

Analysts often assume random components are needed without considering alternate models. In the present study we found that adjustments for within-family correlations were important; however, there was little variability between enumeration districts and random components for enumeration district would be excluded in the most parsimonious model. Similar results indicating small community-level correlations have been found in other studies [4,17] emphasizing the importance of testing the necessity of random components to identify the best model for the data. As hypothesis tests and goodness of fit criteria such as the AIC statistic are straightforward to apply, there is little reason to disregard this advice when using multilevel models to account for multiple sources of correlation.

## Conclusion

Multilevel models are a common tool for examining associations between outcomes and exposures at individual- and group-levels. While hierarchical models are often assumed, many populations do not have a nested data structure. Analysts can employ cross-classified models to allow for non-hierarchical, multilevel data. Researchers can evaluate whether random components are necessary and compare goodness of fit statistics to select a parsimonious model.

Using cross-classified multilevel models we analyzed the association of birth weight with family and community SES while adjusting for numerous potential confounders. The flexibility of cross-classified models allowed us to sample more than one child per family and to include families who moved between births. Results from a restricted to individual-level variables generally found on birth records, showed an association of birth weight with community SES. When we adjusted for a more complete set of individual- and family-level covariates, community SES was not as important. Residual confounding may explain some results previously reported between community SES and birth weight.

## Competing interests

The authors declare that they have no competing interests.

## Authors' contributions

RLY reviewed prior literature, performed statistical analyses, drafted and revised the manuscript, and has approved the final version for submission to this journal. JW, VV, AA, and TFW participated in study design and variable selection, made significant revisions and contributions to the manuscript, and approved the final version for submission to this journal.

## Supplementary Material

Additional file 1 **An additional file including general SAS code to illustrate how cross-classified models can be applied using standard statistical software is included**. Click here for file

## References

[B1] NCHS Vital Statistics of the United States, 1985 Natality 1988 1 Washington D.C.: Public Health Service. U.S. Government Printing Office 88 1113

[B2] Stein A Campbell E Day A McPhearson K Cooper P Social adversity, low birth weight, and preterm delivery British Medical Journal 1987 295 291 293 10.1136/bmj.295.6593.291 3115416 PMC1247138

[B3] O'Campo P Xue X Wang M O'Brien Caughy M Neighborhood Risk Factors for Low Birthweight in Baltimore: A Multilevel Analysis American Journal of Public Health 1997 87 1113 1118 10.2105/AJPH.87.7.1113 9240099 PMC1380883

[B4] Luo Z Wilkins R Kramer M Effect of neighbourhood income and maternal education on birth outcomes: a population-based study Canadian Medical Association Journal 2006 174 1415 1421 Online 10.1503/cmaj.051096 16682708 PMC1455422

[B5] O'Regan K Wiseman M Birth weights and the geography of poverty Focus 1989 12 16 22

[B6] Pickett K Pearl M Multilevel analysis of neighbourhood socioeconomic context and health outcomes: a critical review Journal of Epidemiology and Community Health 2001 1 111 122 10.1136/jech.55.2.111 PMC1731829 11154250

[B7] Coulton C Pandey S Geographic Concentration of Poverty and Risk to Children in Urban Neighborhoods The American Behavioral 1992 35 238 246 10.1177/0002764292035003004

[B8] Pearl M Braveman P Abrams B The Relationship of Neighborhood Socioeconomic Characteristics to Birthwegiht Among 5 Ethnic Groups in California American Journal of Public Health 2001 91 1808 1814 10.2105/AJPH.91.11.1808 11684609 PMC1446884

[B9] Morenoff J Neighborhood Mechanisms and the Spatial Dynamics of Birth Weight American Journal of Sociology 2003 108 976 1017 10.1086/374405 14560732

[B10] Rauh V Andrews H Garfinkel R The Contribution of Maternal Age ot Racial Disparities in Birthweight: A Multilevel Perspective American Journal of Public Health 2001 91 1815 1824 10.2105/AJPH.91.11.1815 11684610 PMC1446885

[B11] Greenland S Principles of multilevel modeling International Journal of Epidemiology 2000 29 158 167 10.1093/ije/29.1.158 10750618

[B12] U.S. Census Bureau Mobility for States and the Nation 1970 Census Reports II

[B13] Simpson GA Fowler MG Geographic Mobility and Children's Emotional/Behavioral Adjustment and School Functioning Pediatrics 1994 93 303 309 8121745

[B14] Rasbash J Browne W De Leeuw J, Kreft I Non-Hierarchical Multilevel Models Handbook of Quantitative Multilevel Analysis 2008 New York: Springer

[B15] SAS Version 9.1 for Windows 2003 Cary, NC: SAS Institute

[B16] Aschengrau A Weinberg J Rogers S Gallagher L Winter M Vieira V Webster T Ozonoff D Prenatal Exposure to Tetrachloroethylene-Contaminated Drinking Water and the Risk of Adverse Birth Outcomes Environmental Health Perspectives 2008 116 814 820 10.1289/ehp.10414 18560539 PMC2430239

[B17] Webster T Hoffman K Weinberg J Vieira V Aschengrau A Community- and Individual-Level Socioeconomic Status and Breast Cancer Risk: Multilevel Modeling on Cape Cod, Massachusetts Environmental health Perspectives 2008 116 1125 1129 10.1289/ehp.10818 18709175 PMC2516595

[B18] Hastie T Tibshirani R Generalized Additive Models 1990 New York: Chapman & Hall/CRC

[B19] Fitzmaurice G Laird N Ware J Applied Longitudinal Analysis 2004 New Jersey: John Wiley & Sons, Inc

[B20] Massachusetts Registry of Vital Records and Statistics 2009 2009 Department of Public Health

[B21] Brooke OG Anderson HR Bland JM Peacock JL Stewart CM Effects on birth weight of smoking, alcohol, caffeine, socioeconomic factors, and pschosocial stress British Medical Journal 1989 298 795 801 10.1136/bmj.298.6676.795 2496859 PMC1836053

[B22] Larroque B Kaminski M Lelong N Subtil D Dehaene P Effects on Birth Weight of Alcohol and Caffeine Consumption during Pregnancy American Journal of Epidemiology 1993 137 941 950 8317451 10.1093/oxfordjournals.aje.a116764

