# Supplementary material for: A multilevel non-hierarchical study of birth weight and socioeconomic status
Source: Int J Health Geogr. 2010 Jul 9;9:36. doi: 10.1186/1476-072X-9-36 (PMC2909180; doi:10.1186/1476-072X-9-36)
Supplement: Additional file 1 — An additional file including general SAS code to illustrate how cross-classified models can be applied using standard statistical software is included. [file 1476-072X-9-36-S1.DOC]

**Additional File 1**

**General Form of Cross-Classified Model in SAS**

The following SAS code can be used to apply cross-classified models.

PROC MIXED data = data1 covtest noclprint;

CLASS cluster1 cluster2 categorical_var;

MODEL outcome = categorical_var continuous_var / chisq solution;

RANDOM intercept / subject = cluster1;

RANDOM intercept / subject = cluster2;

run; quit;

**Notes**

PROC MIXED STATEMENT:

- data1 is a dataset with at least two clustering variables and multiple rows per cluster
- covtest requests a test for non-zero variances
- noclprint suppresses class level information from the SAS output

CLASS STATEMENT:

- clustering variables must be included in a class statement along with any other categorical variables

MODEL STATEMENT:

- the outcome is a continuous, normally distributed variable
- chisq requests chi-square tests to be performed
- solution requests coefficients to be printed

RANDOM STATEMENT:

- cluster1 is a clustering variable (i.e. neighborhood identifier)
- cluster2 is a clustering variable (i.e. family identifier)
- the two random statements request random intercepts for cluster 1 and cluster 2
- if clusters 1 and 2 are not nested, this will provide a cross-classified model

Other options available within PROC MIXED may be applied.
